# Eastern European Fermented Foods: Nutritional Value, Functional Potential, and Cultural Heritage

**DOI:** 10.3390/foods15010028

**Published:** 2025-12-22

**Authors:** Corina-Aurelia Zugravu, Ciprian Constantin

**Affiliations:** 1 Department of Hygiene and Nutrition, “Carol Davila” University of Medicine and Pharmacy, 050463 Bucharest, Romania; 2 Research Metabolism Center—Faculty of Medicine, Titu Maiorescu University, 040051 Bucharest, Romania; ciprian_constantin@yahoo.com; 3 “Carol Davila” Emergency Hospital, Bucharest 1, 88 M. Vulcanescu Street, 010825 Bucharest, Romania

**Keywords:** Eastern Europe, fermented foods, functional foods, probiotics, postbiotics, bioactive peptides, traditional diets, nutrition science

## Abstract

Background: Fermentation is among the oldest and most versatile food processing techniques, enhancing not only shelf life but also nutritional and functional value. While Asian and Western fermented foods are extensively studied, traditional Eastern European fermentations—such as sauerkraut, kefir, bryndza, kvass, and sourdough—remain largely unexplored despite their enduring cultural and dietary importance. These foods combine spontaneous or mixed-culture fermentations, diverse substrates, and unique microbial consortia that may yield distinct bioactive profiles with potential health benefits. Methods: This narrative review synthesizes data from scientific articles, regional reports, and ethnographic sources retrieved from PubMed, Scopus, and Google Scholar up to 2025. Studies were selected for relevance to composition, microbiology, bioactive compounds, and human or experimental health outcomes related to Eastern European fermented foods. Results: Available evidence indicates that traditional fermented dairy, cereal, and vegetable products from Eastern Europe contain fermentation-derived bioactive compounds, including specific bioactive peptides, transformed polyphenols, microbial-synthesized vitamins, organic acids, and live or non-viable microorganisms. Experimental studies describe the generation of ACE-inhibitory peptides, polyphenol biotransformation, and prebiotic or postbiotic metabolites with reported antioxidant, antihypertensive, immunomodulatory, and metabolic effects. However, substantial variability in artisanal production practices and the limited number of standardized human studies currently constrain definitive conclusions. Conclusions: Eastern European fermented foods represent a culturally unique yet scientifically undercharacterized component of functional nutrition. Their complex microbial ecosystems and diverse substrates offer valuable models for studying diet–microbe interactions. Further omics-based and clinical research is warranted to clarify bioavailability, mechanisms of action, and their potential integration into evidence-based dietary strategies.

## 1. Introduction

Fermentation is one of the oldest and most widespread methods of food preservation and transformation, practiced for a long time across different cultures. First evidences of producing fermented beverages are many millennia old [1]. Food fermentation originated in spontaneous biochemical processes in which native microorganisms transform moist substrates through enzymatic activity. Depending on the outcome, this process produces either edible fermented foods or inedible spoiled products. Over time, human societies refined these natural phenomena into controlled techniques, developing regionally specific fermentation systems adapted to local environments and raw materials. The earliest known examples likely involved the alcoholic fermentation of fruits by wild yeasts, while the later emergence of ceramic vessels for storing aqueous foods facilitated broader experimentation and diversification of fermented products.

Beyond its role in extending shelf life and enhancing flavor, fermentation profoundly alters the nutritional and functional profile of foods [2]. Microbial activity during fermentation can enrich products with bioactive peptides, vitamins, organic acids, and secondary metabolites, while also modifying the bioavailability of existing nutrients. These attributes have positioned fermented foods as important contributors to human health and as candidates for functional food development.

In the scientific literature, much of the focus on fermented foods was centered on Asian products (such as miso, kimchi, and natto) or on globally popular Western varieties (such as yogurt, cheese, and sourdough bread). In contrast, the rich tradition of fermented foods in Eastern Europe has received comparatively little attention, despite their cultural and dietary significance. Countries across this region—including Romania, Poland, Ukraine, Russia, and the Balkan states—have long relied on fermentation as a household and artisanal practice to preserve vegetables, dairy, cereals, and beverages. Sauerkraut, kefir, kvass, and a variety of regional cheeses and pickled vegetables are staples that continue to be consumed daily by millions of people. Eastern European fermented foods are of particular interest because they often involve complex microbial consortia, unique raw materials, and traditional preparation methods that can lead to distinctive bioactive profiles. Evidence suggests that such foods may exert beneficial effects on cardiovascular health, gastrointestinal function, immunity, and metabolic regulation [3,4,5]. At the same time, some products carry potential drawbacks, such as high sodium content or variability in microbial quality, which warrant careful evaluation [6]. Eastern Europe has a long-standing reliance on fermented foods that continues to shape both rural and urban dietary patterns. Historically, the region’s continental climate and limited access to fresh produce favored the preservation of vegetables, dairy, and cereals through fermentation, a practice deeply embedded in domestic food culture. Ethnographic and dietary studies document the persistence of these foods—such as sauerkraut, kefir, bryndza, kvass, and borș—as common components of everyday diets in countries including Poland, Romania, Slovakia, and Bulgaria. Reliable quantitative data on the consumption of traditional, homemade fermented foods in Eastern Europe remain scarce, as most products are prepared at the household level and are not captured consistently in national dietary surveys. Therefore, available information reflects broad patterns and cultural significance rather than precise intake figures. While quantitative dietary data remain limited, regional reports and national surveys suggest that fermented products remain nutritionally and culturally significant, contributing to food diversity, microbial exposure, and dietary resilience in contemporary Eastern European populations [7,8,9,10]. Data regarding intake is scattered in already available dietary investigations (see references [7,8,9,10]), and further analysis of dietary intake of fermented products is warranted.

In recent years, there has been a growing interest in gut health, natural foods, and sustainable dietary practices, leading to a renewed interest in classic fermented foods in Eastern Europe. At the same time, scientific output on fermentation, probiotics, and postbiotic mechanisms has expanded rapidly, yet Eastern European products remain underrepresented in comparative studies and European dietary surveys. Contextualizing these foods within current consumer trends and scientific developments is therefore important for understanding their contemporary relevance alongside their historical and cultural significance. Considering the increased consumer interest in fermented foods across Europe, the expanding scientific focus on the human microbiome, and the limited visibility of Eastern European products in the international literature, this review aims to synthesize current knowledge on their composition, microbial ecology, bioactive constituents, and potential health effects.

The general objective is to provide an integrated overview that connects cultural and historical practices with contemporary scientific understanding, highlighting both what is known and the major gaps that remain. Specifically, we examine: (i) the traditional and modern production of Eastern European fermented foods; (ii) their nutritional and microbial characteristics; and (iii) the available experimental and human evidence regarding their functional potential. By situating these foods within the broader European context—both culturally and scientifically—this review seeks to clarify their relevance for consumers, researchers, and public health applications

## 2. Methods—Literature Search Strategy

This narrative review was conducted to provide a comprehensive synthesis of Eastern European fermented foods, their bioactive components, and associated health effects. Relevant literature was identified through searches in PubMed, Scopus, and Web of Science, covering publications up to August 2025. Keywords included combinations of terms such as “fermented foods”, “Eastern Europe”, “sauerkraut”, “kefir”, “kvass”, “fermented dairy”, “fermented vegetables”, “bioactive compounds”, “functional foods”, and “health effects”. In addition to English-language peer-reviewed articles, gray literature, including regional reports, theses, and traditional food compendia in Romanian, Polish, and Russian, was consulted to capture foods and practices underrepresented in mainstream databases. Literature published predominantly in the last two to three decades was considered, with older historical or ethnographic sources included selectively for cultural background.

However, because this is not a systematic review, no predefined time limits or formal inclusion/exclusion criteria were applied. Evidence and its quality were prioritized qualitatively based on scientific relevance, study type (human, animal, in vitro), methodological clarity, and contribution to understanding the microbial, nutritional, or functional characteristics of Eastern European fermented foods, providing a broad overview and critical discussion rather than a quantitative meta-analysis Formal tools (e.g., PRISMA or GRADE), are not applicable to a narrative synthesis. In the end, the publications included span in vitro studies, a smaller number of animal studies, and limited human observational or intervention studies, supplemented by historical and cultural descriptions to contextualize the functional potential of these foods.

## 3. Historical and Cultural Context of Classes of Fermented Foods

Fermentation has been an integral part of Eastern European food culture for centuries, serving as both a necessity for preservation and a cornerstone of culinary identity. The long, harsh winters, limited access to fresh produce during colder months, and the abundance of raw materials such as cabbage, milk, rye, and root vegetables created conditions in which fermentation was not only practical but indispensable. Generations of households developed fermentation practices that were deeply embedded in daily life, transmitted through family traditions, and often tied to seasonal cycles and local festivities. Traditional fermentation procedures generally rely on spontaneous microbial activity initiated by naturally occurring microorganisms present on raw materials, utensils, or in the surrounding environment. The process typically involves minimal technological intervention and is conducted under ambient conditions that favor the growth of desirable bacteria, yeasts, or molds [11]. Such fermentations are often performed in small batches using household or community-based practices, where environmental factors and indigenous microflora impart characteristic sensory and nutritional properties to the final products [12].

Eastern European fermented foods encompass a diverse array of products, reflecting regional biodiversity, cultural traditions, and resource availability. These foods can be broadly categorized into dairy, vegetables, cereals, and beverages, each with distinctive microbial communities, bioactive compounds, and potential health benefits.

### 3.1. Vegetable Fermentation

Vegetable fermentation is perhaps the most iconic expression of this tradition. Cabbage, preserved as sauerkraut, became a dietary staple across Central and Eastern Europe. The product was known by different names, according to each region [13]. In Romania, Poland, Ukraine, and the Balkans, large barrels of shredded cabbage or cabbage leaves prepared in autumn [14] were relied upon to provide essential vitamins throughout the winter, when scurvy and micronutrient deficiencies were otherwise a risk. The quantity of cabbage in a household was either a sign of prosperity or of scarcity. Traditional sauerkraut fermentation is dominated by lactic acid bacteria, mainly Leuconostoc, Lactobacillus, and Pediococcus species, which succeed sequentially during fermentation. As boosters for preservation, many other plants are added (mustard, celery, leaves, horseradish, etc.), contributing to the enrichment of the nutritional value of the fermented product.

Sauerkraut is not merely a side dish but a foundation for numerous cooked meals, symbolizing resilience in the face of food scarcity. Bell peppers, cucumbers, carrots, garlic, beets and even small watermelons are also frequently fermented, producing pickles [15], each with different microbial communities and flavors that became part of everyday diets. These products illustrate how local biodiversity and microbial ecology intersect with nutritional needs [10,16].

The microbial diversity of fermented vegetables is highly variable and influenced by preparation methods, local microflora, and salt concentration, creating unique microbial “signatures” that differentiate regional products [17].

### 3.2. Fermented Dairy Products

Fermented dairy products hold a similarly prominent role in Eastern European traditions. Although some products discussed (e.g., Matzoon, Ayran) originate beyond strict geopolitical definitions of Eastern Europe, they are included due to their long-standing integration into Eastern and Southeastern European food traditions through historical, cultural, and culinary exchange.

Kefir, originating in the Caucasus, became widespread in Russia and Eastern Europe by the 19th century, and was celebrated for its purported health benefits long before the mechanisms of probiotics and lactic acid bacteria were understood. Kefir grains contain a stable symbiotic consortium of lactic acid bacteria (mainly *Lactobacillus* spp., *Lactococcus* spp., and *Leuconostoc* spp.), acetic acid bacteria, and yeasts (such as *Saccharomyces* and *Kluyveromyces* species), creating a beverage rich in nutrients. The fermentation process improves protein digestibility and produces naturally occurring antimicrobial compounds [18].

Other traditional dairy beverages, such as ryazhenka in Russia (fermented baked milk) or ayran in the Balkans (a yogurt-based drink), highlight the creativity with which dairy was preserved and consumed. Their production illustrates regional adaptations, where local processing (heat treatment, dilution, mixing with herbs or salt) shaped both sensory properties and nutritional profiles. Ryazhenka is a baked fermented milk, traditionally prepared by heating milk before fermentation. This process yields Maillard reaction products, mild acidity, and a unique flavor profile, with potential antioxidant properties [19]. Ayran, a diluted yogurt-based beverage, is widely consumed in the Balkans. Its hydrating properties and probiotic potential make it an important functional food in both traditional and modern diets [20]. Domestic Bulgarian yogurt, “kiselo mlyako”, is produced similarly to ayran, but has a historical recognition of having antiaging effects. At the start of the 20th century, the renowned Russian scientist and Nobel laureate Ilya Mechnikov (1845–1916) turned his attention to the issue of human aging. Mechnikov identified Bulgarian yogurt (“kiselo mlyako”) as a food capable of suppressing these harmful bacteria that cause ageing. Based on this, he argued that Bulgaria’s unusually high number of centenarians was linked to the regular consumption of this traditional yogurt [21,22].

Matzoon or matsuni is a type of traditional Armenian and Georgian fermented dairy product that consists of milk and a starter culture or previously made matzoon. It is typically made with boiled cow’s milk combined with yogurt, and the mixture is then allowed to sit in a warm place until it becomes homogenous. Semi-firm and with a distinctive, tangy flavor, matzoon is featured in numerous traditional Armenian dishes and beverages, both sweet and savory, such as the refreshing beverages known as matsnabrdosh and tahn, various sauces for dishes made with local greens, and soups [23].

Cheese-making traditions also flourished, especially in areas with wide pastures, with products such as bryndza (a sheep’s milk cheese popular in Romania and Slovakia) representing both nutritional security and cultural identity.

Cheeses such as bryndza and other sheep- or cow-milk varieties undergo lactic fermentation, which develops texture, flavor, and bioactive peptides. These cheeses also provide a concentrated source of nutrients in regions with limited access to fresh produce during winter months. These products highlight the dual function of fermentation as both nutrient preservation and functional enrichment [24].

### 3.3. Cereal-Based Fermentations

Cereal-based fermentations are another cornerstone of Eastern European food traditions. Fermentation of cereals is a long-standing practice in the region, primarily for bread and beverages. Rye sourdough bread has been a staple across the Baltic states, Poland, and Russia, not only for its flavor but also for its longer shelf life and enhanced digestibility. Fermentation of dough ameliorates digestibility, increases mineral bioavailability, and generates bioactive peptides. The long fermentation also contributes to lower glycemic indices compared to industrial breads, suggesting potential benefits for metabolic health [25,26].

Other cereal-based fermentations include fermented porridges, like kissel [27] and regional sourdough variations, often consumed as staple meals or snacks [28,29], highlighting the adaptability of fermentation to local grains and seasonal availability. A wide range of cereal-based fermented beverages is also consumed across Eastern Europe and is discussed in the following subchapter.

### 3.4. Fermented Beverages

Fermented beverages in Eastern Europe extend beyond dairy and cereal-based products, representing a broad category with unique microbial and bioactive properties [30].

Kvass remains the most widespread traditional beverage. It is a lightly alcoholic beverage made from fermented rye or fermented bread, emblematic of Slavic food culture, mentioned for the first time in the 10th century. Other grains like barley and wheat, or fruits and vegetables, can also be used. Historically considered a “drink of the people”, it was consumed daily across social classes, providing hydration, modest nutrition, and safe microbial quality in times when water could be unreliable. Traditionally consumed across social classes, it has served both as a nutritional supplement and as a culturally symbolic beverage. It involves fermenting the ingredients with water, sugar, and yeast, resulting in a lightly alcoholic beverage with a sour, tangy taste and natural carbonation. Popular types include malty bread kvass, earthy beet kvass (often used in borscht), and even lettuce kvass [31,32,33].

Similar cereal-based fermented beverages were prepared regionally, sometimes flavored with herbs, honey, or berries, reflecting the biodiversity of local environments. Fermented herbal or berry infusions, such as elderberry or cranberry ferments, are common in rural areas, often prepared in households for seasonal consumption. Even in big cities, elderberry fermented beverages (“socata” in Romanian) are prepared in season by many families, as a healthier alternative to commercial soft drinks.

“Borș” is a traditional Romanian fermented liquid, distinct from the beetroot-based borscht of Ukraine and Russia. It is prepared from wheat or barley bran, corn flour, and water, often with the addition of a slice of bread—particularly black bread—to initiate fermentation. The mixture undergoes natural fermentation driven by lactic acid bacteria and yeasts, producing a tangy, slightly yellow liquid with a refreshing, mildly pungent aroma, eventually enriched with aromatic herbs (e.g., lovage). In Romanian cuisine, “borș” is primarily used to impart a characteristic sour flavor to “ciorbe” (sour soups), but it can also be consumed directly as a potential probiotic beverage. The fermented solids can be dried and preserved as a starter, known as “huște”, for subsequent batches. Nutritionally, “borș” is rich in lactic acid, vitamins, and minerals derived from the cereal bran, and it contains phenolic compounds that confer antioxidant properties. Conceptually, borș is similar to Russian kvass—a fermented rye bread drink—but it remains unique in its composition, preparation, and culinary application [34,35].

Braga, boza or busa are traditional fermented cereal beverages widely consumed in the Balkans, Turkey, and also in many parts of Eastern Europe [36]. The names and recipes vary by region—“braga” and “busa” are used in specific areas—reflecting local cereal availability and fermentation practices. This diversity not only influences taste and texture but also affects the nutritional and microbial profiles, illustrating the intersection of culture, diet, and health in Eastern European fermented foods. They are typically prepared from a mix of cereals such as wheat, maize, millet, barley, oats, rye, or rice, which serve as a source of carbohydrates, dietary fiber, and essential micronutrients. The fermentation process is driven by LAB and yeasts. LAB produces organic acids, primarily lactic acid, which lower the pH, preserve the beverage, and contribute to its characteristic tangy flavor. Yeasts generate low levels of ethanol and carbon dioxide, adding mild effervescence. Fermentation also enhances the nutritional value of the drink [37].

Kombucha, though originally from East Asia, became integrated into Russian and Eastern European traditions by the 19th and 20th centuries. It s consumption gained popularity due to its perceived health benefits, safety, and appealing sensory characteristics [38]. Its fermentation produces organic acids, vitamins, and a diverse microbial consortium [39]. While a borderline case in terms of origin, kombucha exemplifies the region’s capacity to adapt and integrate fermented beverages into everyday diets [40].

Across all categories, Eastern European fermented foods share several characteristics. First, we notice the microbial diversity. Spontaneous or starter-driven fermentation generates complex communities of bacteria and yeasts, producing bioactive metabolites. Fermentation increases bioavailability of vitamins, minerals, and proteins while producing bioactive peptides and organic acids [41,42]. All of them carry a functional potential. Evidence suggests health benefits. Even more, these fermented foods are eaten daily by a high number of people, due to their cultural embeddedness. These foods are integral to seasonal diets, traditions, and social rituals, which helps maintain their consumption and microbial authenticity.

The cultural dimension of fermentation in Eastern Europe goes far beyond preservation. These foods often carried symbolic and ritual meaning. In Romania, sauerkraut leaves are essential for preparing sarmale (cabbage rolls), a dish associated with weddings, holidays (Christmas, Easter), and community gatherings. Sarmale is frequently prepared in large quantities for festive occasions, and fermented cabbage (sauerkraut) is often used for its flavor and traditional wrapping in many regions [43]. During fasting periods of Orthodox Christianity—such as Lent or the Nativity Fast—plant-based foods become central, and fermented vegetables like sauerkraut are used because they are allowed and because they provide nutrients when fresh vegetables are less available [44]. Sarmale wrapped in sauerkraut leaves can also be adapted (meatless or with mushrooms) during fasting [45]. In Russia, kvass has been deeply embedded in culture for over a millennium. Its origin dates back at least to the Primary Chronicle of Kievan Rus (989 AD), and it was consumed by people of all social classes. It was offered to guests as a gesture of hospitality, served in communal settings, during festivals, markets, celebrations, and sometimes used in religious fasts or daily meals [46]. As for kefir, traditional narratives from the Caucasus note that kefir grains were zealously guarded cultural treasures, passed from generation to generation. The symbiotic culture (bacteria + yeasts) was considered precious, with households maintaining their own grains as heirlooms [47].

Industrialization in the 20th century altered the production of many of these foods, shifting from household to factory-scale processes. Yet, unlike in some Western contexts, homemade fermentation remained widespread in Eastern Europe, partly due to strong cultural attachment, partly due to economic necessity. Even today, urban households frequently prepare their own sauerkraut or pickles, preserving continuity with ancestral practices [48,49]. This persistence has ensured that microbial diversity, traditional methods, and cultural symbolism remain alive, making Eastern European fermented foods unique not only in their composition but also in their sociocultural significance.

In recent decades, scientific and consumer interest has converged on these traditional foods. Researchers are beginning to uncover the bioactive compounds [50] and microbial profiles [51] that are responsible for their health-promoting effects, while consumers are increasingly drawn to them as “authentic” and “natural” dietary components [52]. Understanding their historical and cultural roots is therefore essential, not only for appreciating their continued role in the daily diet but also for framing them as potential functional foods within contemporary nutrition science.

## 4. Bioactive Compounds and Functional Properties

This section integrates microbiological, nutritional, and functional aspects of Eastern European fermented foods, focusing on representative examples while avoiding repetition of general fermentation mechanisms discussed elsewhere. Eastern European fermented foods are rich in a variety of bioactive compounds that emerge from microbial activity during fermentation, interactions with the food matrix, and enzymatic transformations of native substrates. These compounds confer functional properties, contributing to cardiovascular, gastrointestinal, metabolic, and immune health.

### 4.1. Bioactive Peptides

Bioactive peptides are short chains of amino acids (often 2–20 residues, though sometimes longer) generated during proteolysis—i.e., the enzymatic breakdown of larger proteins—by fermenting microorganisms. These peptides may exert diverse physiological effects such as ACE-inhibition (important for blood pressure control), antioxidant activity (scavenging reactive oxygen species), immunomodulation, and antimicrobial effects. Their production, stability, and bioactivity are strongly influenced by the protein substrate (type and content), length and conditions of fermentation, and the microbial community involved (species, strain, and their proteolytic systems) [53]. Several peptides with known or potential physiological effects have been identified in traditional Eastern European fermented foods (Table 1).

#### 4.1.1. Bioactive Peptides in Dairy Products

One of the most studied products is kefir. In kefir, recent studies show that the presence and activity of *Lactobacillus plantarum* and other lactobacilli in fermentation significantly increase ACE-inhibitory activity over time. For example, one kefir sample fermented with *L. plantarum* showed ~87.3% ACE inhibition after an extended fermentation (28 days), with an IC_50_ of ~19.7 mg/L. While the specific peptide sequences were not identified in this study, earlier proteomic analyses of kefir have reported the presence of β-casein–derived peptides such as Val–Pro–Pro (VPP) and Ile–Pro–Pro (IPP), both recognized ACE inhibitors [55,62,63]. Lactotripeptides from casein fragments (Val–Pro–Pro, Ile–Pro–Pro) are among the best documented in kefir and yogurt-like fermentations [64,65]. Peptides from αs1-casein and β-casein with antioxidant and antimicrobial activities have also been isolated in kefir-like LAB fermentations (e.g., *Lactobacillus helveticus*, *L. plantarum*) [54,66].

Bryndza cheese exhibits substantial proteolysis during ripening. A study on the effects of partial replacement of salt with potassium chloride in bryndza found that changes in protein breakdown (proteolytic patterns) are observed, suggesting that the peptides produced during these processes may vary in abundance and composition [67]. The microbiota of bryndza (and its bacterial/yeast communities) have already been characterized, showing rich diversity that is a precondition for diverse proteolytic activity, resulting potentially in active peptides being generated. For example, *Lactobacillus paracasei*, *Lactococcus lactis*, and other lactic acid bacteria are commonly present in bryndza [68,69].

Thus, one can conclude that kefir and bryndza cheese contain peptides that have demonstrated ACE-inhibitory activity in vitro and in animal models [58,70]. These peptides may contribute to cardiovascular benefits traditionally associated with fermented dairy consumption [57,71,72,73], but human studies are still needed.

#### 4.1.2. Bioactive Peptides in Cereal Products

Sourdough fermentations of various cereal flours (whole wheat, spelt, rye, kamut) with selected lactic acid bacteria have been shown to produce antioxidant peptides. In one study, extracts from sourdoughs had significantly higher radical scavenging activity compared to chemically acidified doughs. The researchers identified 25 specific peptides (8 to 57 amino acids in length) via LC-MS/MS that were stable even after simulated digestive enzyme hydrolysis [74]. Studies have shown that water/salt-soluble extracts from sourdoughs possess significantly higher radical scavenging activity compared to extracts from chemically acidified doughs [59,60,61].

Rye sourdough bread has repeatedly been shown to improve postprandial glucose and insulin responses when compared to non-sourdough breads—while in many of these studies the precise peptides are not fully identified, the improved metabolic responses are believed to be partly mediated by peptides and organic acids released during fermentation [75,76]. This has been supported by EFSA’s scientific opinions on rye bread and other clinical trials with sourdough rye crispbread [77]. The fermentation of sourdough breaks down gluten and phytic acid, potentially releasing minerals and altering the bread’s structure to create a lower and more sustained release of glucose into the bloodstream [78]. One of the causes of the mentioned effects is a longer fermentation, which tends to allow more extensive proteolysis, generating a broader spectrum of peptides. The kefir example above also showed increasing ACE inhibition with longer incubation [58].

Another factor that enhances the peptides production is the microbial composition. Strains differ in protease sets; e.g., *L. plantarum* in kefir gave higher ACE-inhibition. In cereals, mixed LAB communities produce more diverse peptide profiles than single-strain fermentations. When a mixed community of microorganisms, such as multiple LAB strains, is used for fermentation (like in cereals), it leads to a wider range of peptide products. The interaction between different microbial strains in a mixed culture can be synergistic, enhancing the overall production of diverse peptides compared to using a single strain [79].

Last but not least, peptide production depends on the substrate protein content and quality [80]. Dairy is rich in casein and whey proteins; cereals have prolamins, glutelins, and other storage proteins (with lower digestibility). The type of cereal (rye, wheat, spelt) and the level of protein affect how many peptides and which ones can be released. Wholegrain flours or intact kernels often result in more substrates for proteolysis, which explains the presence of multiple peptides in sordough rye bread.

Although in vitro and animal models provide clear evidence for ACE-inhibitory and antioxidant peptides in fermented dairy and cereals, human data on peptide-specific clinical endpoints and bioavailability are limited; this issue is discussed in detail in Section 7 (Challenges and Research Gaps).

### 4.2. Polyphenols and Secondary Metabolites

Polyphenols and other secondary plant metabolites (flavonoids, phenolic acids, betalains, glucosinolates and derivatives) are major contributors to the antioxidant, anti-inflammatory and signaling properties attributed to many plant-based foods [81,82,83]. Fermentation of vegetables, cereals, and berries can significantly alter the content, chemical form, and bioavailability of polyphenols, flavonoids, betalains and related secondary metabolites. In traditional Eastern European fermentations such as sauerkraut, beet kvass and berry ferments, these compounds may be liberated from bound forms or transformed by microbial enzymes, altering antioxidant potential. These polyphenols and their metabolites can act as free radical scavengers, modulate the gut microbiota, and influence inflammatory and metabolic pathways [84]. The following subsections detail the underlying biochemical mechanisms, examples from vegetable and cereal fermentations, and implications for health. Key transformations of polyphenols and related secondary metabolites in traditional Eastern European fermented foods are summarized in Table 2.

Microbial fermentation affects polyphenols through several, well-characterized biochemical routes:

- Hydrolysis of ester/ether bonds and depolymerization: microbial esterases and glycosidases cleave polyphenol conjugates (e.g., glycosides, phenolic acid esters), liberating aglycones and free phenolic acids that are more extractable and sometimes more bioactive [84,85];
- Decarboxylation and reduction: some LAB possess phenolic acid decarboxylases (pdc) and related enzymes that convert hydroxycinnamic acids (e.g., ferulic, p-coumaric acid) into vinyl/ethyl derivatives with altered bioactivities. Strain-level differences in pdc genes materially affect the metabolic outcome [86,87,88];
- Deglycosylation: microbial β-glucosidases remove sugar moieties from flavonoid glycosides, producing [84];
- Formation of novel metabolites (postbiotic phenolics): microbial metabolism can produce smaller phenolic catabolites and short-chain phenolic acids that may exert direct biological effects or act via the gut microbiota [84,89].

These mechanisms also explain the substantial variability observed between studies.

#### 4.2.1. Polyphenols and Secondary Metabolites in Vegetable Fermentations (Sauerkraut, Beet Kvass, Mixed Ferments)

- Sauerkraut and brassica ferments: Lactic acid fermentation of cabbage alters its phytochemical profile in predictable ways: glucosinolates may be hydrolysed to isothiocyanates or nitriles depending on processing (cutting, salting, temperature) [90], and polyphenol extractability can increase as cell walls are partially degraded and conjugates hydrolysed [91]. Targeted metabolomic and metagenomic studies of sauerkraut document dynamic shifts in metabolites and microbial functions across fermentation stages, linking specific microbial succession to secondary-metabolite transformations. These changes underlie measured increases in certain antioxidant activities in many (but not all) sauerkraut preparations [92,93].
- Beet kvass and betalain-rich ferments: Beetroot fermentations (beet kvass) present a slightly different profile: the dominant pigments are betalains (betacyanins and betaxanthins), not flavonoids [94]. Several recent analyses of commercially produced and traditional fermented beetroot juices in Polish markets show high antioxidant capacity and measurable betalain content in fermented products, although thermal processing and fermentation conditions influence betalain stability. Some studies report retention or even improved antioxidant activity in certain beet ferments, while others show losses depending on boiling/processing and long fermentation times—so product formulation and process control matter [95,96].

Overall, vegetable fermentation alters polyphenol and glucosinolate profiles and may increase extractability, depending on process conditions [97].

#### 4.2.2. Polyphenols and Secondary Metabolites in Cereal Fermentations (Rye Sourdough and Related Products)

Rye and other cereal sourdough fermentations illustrate another common pattern: many phenolic compounds in cereals are bound (esterified to cell walls, ferulic acid conjugates). LAB and fungal enzymes during sourdough fermentation can release these bound phenolics (e.g., ferulic, vanillic, p-coumaric acids), increasing extractable phenolics and antioxidant capacity of the dough and bread [85]. Metabolic and peptidomic studies on sourdoughs (wheat and rye) demonstrate strain-dependent metabolism of phenolic acids and increased extractability after fermentation, which contributes to improved in vitro antioxidant markers and may partly explain lower postprandial glycemic responses of long-fermented rye breads [98]. However, the magnitude of these changes depends on flour type, bran fraction, fermentation length, and the specific LAB/yeast strains used [98,99].

#### 4.2.3. Polyphenols and Secondary Metabolites in Fermented Beverages (Kombucha and Tea-Based Ferments)

Kombucha and similar tea-based ferments derive most polyphenols from the tea substrate (catechins, theaflavins, flavonols). Fermentation by the SCOBY (yeasts + bacteria) modifies the polyphenolic profile: some polyphenols are transformed (deglycosylated, oxidized), and the total measured antioxidant activity often increases relative to the starting infusion, particularly when tea residues or polyphenol-rich adjuncts are used [100]. Recent studies show that formulation (type of tea, addition of herbs) and fermentation time markedly influence total phenolics and antioxidant measures. However, human clinical evidence remains limited, and safety and variability warrant cautious interpretation [97,101].

#### 4.2.4. Factors Driving Heterogeneity in Outcomes

Several variables explain why studies report increases, no change, or decreases in polyphenol content after fermentation:

- Raw material and pre-processing: Cooking, blanching, or boiling before fermentation can leach or degrade heat-sensitive compounds (notably vitamin C and some polyphenols/betalains). Studies show boiling + fermentation often reduces some phenolics compared with raw [96,102].
- Microbial strains and enzymatic repertoire: Presence/absence of β-glucosidase, esterases, and pdc genes determines whether bound phenolics are liberated or decarboxylated into less extractable forms. Strain selection (or spontaneous flora) is therefore critical [85,89].
- Fermentation time, temperature, and oxygen: Longer or warmer fermentations often increase conversion but may also permit oxidative degradation; controlled low-temperature fermentations can preserve labile pigments [92,96].
- Matrix interactions: Polyphenols can bind to proteins or fiber; proteolysis and polysaccharide breakdown during fermentation alter binding equilibria and extractability [84].

#### 4.2.5. Implications for Bioavailability and Health

Increased extractability is a plausible first step toward improved bioavailability, but human absorption and metabolism depend on intestinal and hepatic processing as well as gut microbiota conversion to smaller phenolic metabolites. Few studies combine food-level fermentation analysis with human pharmacokinetic or clinical endpoints; this remains a key evidence gap [84,99]. Multiple in vitro and animal studies show fermentation-enhanced antioxidant and anti-inflammatory activities; however, direct clinical evidence that fermented vegetable or sourdough-derived polyphenols reduce disease endpoints is limited and heterogeneous. Hence, claims should be framed as potential functional contributions supported by mechanistic data rather than proven clinical effects [84,92]. Paired studies that couple detailed fermentation metabolomics (untargeted LC-MS) with human absorption (plasma metabolomics) and clinical endpoints are still rare but needed [92,99]. Strain-level functional screening for key enzymes (β-glucosidase, esterases, pdc) and deliberate starter selection could standardize beneficial transformations while preserving traditional sensory profiles [85,89]. We noticed that process optimization (time/temperature/salt) studies to maximize retention of labile polyphenols (e.g., betalains) while promoting liberation of bound phenolics are essential for product development [96].

**Table 2:** Polyphenols and secondary metabolites in traditional Eastern European fermented foods and their microbial transformations.

Food	Major Polyphenol/Pigment Classes	Microbial or Enzymatic Transformation	Reported Outcome	Key References Cited
Sauerkraut	Phenolic acids, flavonoids, glucosinolates	β-glucosidase-mediated deglycosylation; partial hydrolysis of glucosinolates	↑ extractable phenolics, ↑ antioxidant activity	[84,103]
Beet kvass	Betalains (betacyanins, betaxanthins), phenolic acids	LAB fermentation preserves betalains, produces phenolic catabolites	Mixed outcomes; potential ↑ antioxidant potential	[95]
Rye sourdough	Ferulic, p-coumaric, vanillic acids (bound)	LAB esterase and decarboxylase activity releases bound phenolics	↑ extractable phenolics, improved antioxidant capacity	[59]
Kombucha	Catechins, theaflavins, flavonols	Yeast/bacterial oxidation and deglycosylation	↑ total phenolics (moderate), altered flavonoid profile	[100]

### 4.3. Probiotics, Prebiotics and Postbiotics

Fermented foods represent complex microbial ecosystems containing live probiotic organisms and a rich milieu of bioactive microbial metabolites, collectively referred to as postbiotics. Their effects on health are discussed in Section 5.2 and Section 5.3. Unlike isolated probiotic supplements, traditional fermented foods deliver microorganisms within a nutrient-rich matrix, which can modulate their survival, colonization, and host interactions.

#### 4.3.1. Probiotic Microorganisms in Eastern European Fermentations

Traditional Eastern European fermentations rely largely on LAB and, to a lesser extent, yeasts, many of which meet the FAO/WHO definition of probiotics when administered in adequate amounts.

Dominant genera include:

- *Lactobacillus* (especially *L. plantarum*, *L. brevis*, *L. casei*, *L. helveticus*, *L. kefiri*).
- *Leuconostoc mesenteroides* and *Leuconostoc lactis*.
- *Lactococcus lactis* and *Pediococcus pentosaceus*.
- Yeasts such as *Saccharomyces cerevisiae* and *Kluyveromyces marxianus*.

These taxa are recurrently identified in kefir, sauerkraut, bryndza, and rye sourdough and are associated with distinct health-related mechanisms, as presented in Table 3.

Recent human trials support that regular consumption of fermented foods increases microbiota diversity and reduces systemic inflammatory markers (e.g., IL-6, TNF-α) in healthy adults [105]. These effects were stronger for multi-strain, naturally fermented foods such as kefir and sauerkraut than for single-strain probiotic products [51].

#### 4.3.2. Pre- and Postbiotic Compounds in Eastern European Fermentations and Their Functional Roles

Even in the absence of viable cells, fermented food matrices remain biologically active due to a broad range of pre- and postbiotic compounds. Prebiotics are substrates that are selectively utilized by host microorganisms, conferring a health benefit; postbiotics are preparations of inanimate microorganisms and/or their components that confer a health benefit. In the context of traditional Eastern European fermented foods, both classes are important: fermentation can generate or concentrate fermentable substrates (prebiotics), and it produces or releases microbial-derived molecules and structural components (postbiotics) that act on host physiology even when microbes are non-viable.

- Prebiotics:

The key compound classes of prebiotics are fermentable oligosaccharides (including arabinoxylo-oligosaccharides from cereals), resistant starch fractions, pectic-oligosaccharides, and certain microbial exopolysaccharides (EPS) produced in situ [110]. Sources in Eastern European ferments are different in terms of function of the substrate. In rye sourdough and other cereal ferments, partial hydrolysis of hemicelluloses and arabinoxylans during long fermentation can release short-chain oligosaccharides (AXOS) that are fermentable by beneficial colonic bacteria [111]. In vegetable ferments (sauerkraut, mixed pickles) there is cell-wall breakdown and endogenous enzyme action during fermentation that may increase the proportion of soluble fibers and pectic-oligosaccharides, potentially improving fermentability [112]. As for dairy ferments (kefir), certain EPS (e.g., kefiran) synthesized by kefir-associated microbes resist host digestion and can act as substrates for gut microbes [113]. The mechanisms of action of prebiotics include selective stimulation of beneficial microbial taxa (e.g., *Bifidobacterium*, certain *Faecalibacterium* spp.), leading to increased production of SCFAs in the colon [114], indirect modulation of host physiology via SCFAs (energy source for colonocytes, signaling via G-protein coupled receptors such as GPR41/43, epigenetic modulation via histone deacetylase inhibition [115] and improvement of mineral bioavailability [41]. Most mechanistic data for these effects come from in vitro fermentation models and animal studies [116,117,118]; human trials directly linking specific fermentation-generated prebiotics from traditional foods to clinical endpoints are limited [119]. Demonstration of selective utilization and downstream SCFA changes in human feeding studies is an important research priority.

- Postbiotic compounds produced or enriched by fermentation belong to SCFAs, organic acids (lactic, acetic), microbially released bioactive peptides, vitamins (microbial-synthesized B-vitamins, menaquinones), microbial cell-wall components (peptidoglycan fragments, lipoteichoic acids), heat-stable metabolites, and extracellular vesicles [120].

SCFAs and organic acids are produced during fermentation and during subsequent colonic metabolism. Many traditional ferments contain measurable lactic and acetic acids; some cereal and vegetable ferments may contribute substrates that increase colonic SCFA production. Microbial peptides and small molecules result from proteolysis during dairy and cereal fermentation, releasing peptides (see Section 4.1) that can act locally or systemically (e.g., ACE-inhibitory peptides). The structural microbial components are sterilized (cell-free) fractions from sauerkraut and some dairy ferment supernatants. They retain biological activity in vitro and in vivo, indicating that non-viable cell material and soluble metabolites contribute to effects attributed to the food matrix [121,122,123]. The mechanisms of action of postbiotics are immune modulation- cell wall components and bacterial metabolites interacting with pattern recognition receptors (e.g., toll-like receptors-TLRs, Nucleotide-binding Oligomerization Domain proteins -NODs) on epithelial and immune cells [124], modulating cytokine production and mucosal immune tone, barrier function, where SCFAs and certain microbial metabolites enhance expression/localization of tight-junction proteins (occludin, claudins), reduce epithelial permeability, and promote mucin production [125]. There are also antimicrobial and competitive effects: organic acids and bacteriocins in cell-free supernatants inhibit pathogen growth and can alter luminal ecology without requiring live cells [126]. SCFAs also act as signaling molecules, influencing metabolic and immune health [127].

Preclinical studies (cell culture, gnotobiotic and conventional animal models) provide mechanistic support for many postbiotic actions [125,128]. Human evidence is emerging: randomized feeding studies with whole fermented foods have shown changes in biomarkers of inflammation, gut permeability, and metabolite profiles, but disentangling the contributions of live microbes, prebiotic substrates, and postbiotic molecules remains challenging [105,129,130,131]. Isolated postbiotic preparations (e.g., heat-killed strains, purified EPS) are less commonly tested in large human trials but show promise in controlled settings [132,133,134,135].

### 4.4. Vitamins and Micronutrients in Eastern European Fermentations

Fermentation can increase or stabilize the content of several vitamins and minerals. Certain lactic acid bacteria and yeasts are capable of synthesizing B-group vitamins, including riboflavin, folate, and cobalamin (vitamin B_12_), during dairy, cereal, and vegetable fermentations [136]. For example, *L. plantarum* and *L. fermentum* strains used in sourdough and sauerkraut are known folate producers, while Propionibacterium freudenreichii in some cheeses contributes to B_12_ and vitamin K_2_ (menaquinone) formation—nutrients important for hematologic, bone, and cardiovascular health [137,138]. In parallel, microbial metabolism during fermentation can improve mineral bioavailability. Organic acid production lowers pH and reduces antinutritional factors such as phytates and oxalates, which normally chelate iron, zinc, calcium, and magnesium. The degradation of phytic acid in fermented cereals and legumes markedly enhances the absorption of these minerals, while lactic acid itself may facilitate passive mineral transport in the intestine [139,140,141]. Although much of the evidence arises from compositional and in vitro studies, emerging human trials suggest that long-term inclusion of fermented foods can modestly improve micronutrient status and overall dietary quality [142].

### 4.5. Organic Acids in Eastern European Fermentations

Organic acids—mainly lactic, acetic, propionic, and butyric acids—are among the most characteristic metabolites produced during fermentation by lactic acid bacteria and associated yeasts. These acids are central to both the safety and sensory profile of fermented foods [143], contributing to flavor development, texture, and extended shelf life through pH reduction and inhibition of spoilage organisms. Beyond preservation, organic acids exert nutritional and physiological roles. Lactic and acetic acids can enhance mineral bioavailability [144]. Some organic acids also contribute to metabolic regulation [145,146]. In traditional Eastern European products such as sauerkraut, kefir, and kvass, organic acids are key indicators of successful fermentation and product stability.

The health-promoting potential of fermented foods arises not from a single compound but from multiple interactions between components, making traditional foods functionally complex, potentially providing broader health effects than isolated bioactive compounds or supplements [147,148,149].

## 5. Health Effects: Evidence Overview

Eastern European fermented foods contain diverse bioactives and microbes (see Section 4) that plausibly impact different health outcomes. The subsections below summarize mechanistic evidence and human studies for each domain.

### 5.1. Cardiovascular Health

Fermented dairy, particularly kefir and traditional cheeses, and fermented vegetables like sauerkraut, may contribute to blood pressure regulation, lipid modulation, and vascular function.

The antihypertensive and vascular effects observed in vivo are thought to reflect the combined action of ACE-inhibitory peptides (see Section 4.1) and antioxidant polyphenols (see Section 4.2), which protect endothelial cells and modulate nitric oxide availability. LAB fermentation can produce bioactive lipids, including conjugated linoleic acid, which may influence lipid metabolism [150,151]. A randomized trial with kefir consumption (200–500 mL/day for 8–12 weeks) in hypertensive patients demonstrated modest but significant reductions in systolic and diastolic blood pressure [3]. However, a recent meta-analysis of RCTs found no significant effect of kefir on blood pressure in adults [152]. In vitro studies of bryndza and other sheep-milk cheeses confirm ACE-inhibitory activity and antioxidant capacity [153]. Observational data from Eastern European populations consuming fermented vegetables suggest an association with lower cardiovascular risk markers, although controlled studies remain limited [9].

### 5.2. Gastrointestinal Health

Traditional Eastern European fermented foods such as kefir, sauerkraut, and rye sourdough exert beneficial effects on gastrointestinal function through their combined content of live microorganisms, fermentable substrates, and bioactive metabolites. These foods enhance gut microbial diversity, competitively inhibit pathogenic bacteria, and strengthen intestinal barrier integrity via lactic acid bacteria and yeasts [9,147]. Fermentation metabolites—particularly SCFAs—serve as energy substrates for colonocytes and regulate mucosal immune responses, while polyphenols from fermented vegetables and cereals might act as prebiotic substrates promoting the growth of beneficial taxa, though they are not recognized prebiotics sensu stricto. Clinical observations report that kefir and sauerkraut consumption can improve stool frequency and consistency [154] in individuals with functional constipation [155], while small-scale studies suggest a reduction in Helicobacter pylori load with regular fermented vegetable intake [156]. Animal models further support improved barrier function and anti-inflammatory signaling following fermented dairy and cereal consumption [157,158], although large-scale human trials remain limited [157,159] and preliminary.

### 5.3. Immune Function

In Eastern European diets, traditional fermented foods such as kefir, bryndza, and fermented vegetables may influence both innate and adaptive immune responses through microbial metabolites and bioactive compounds. Lactic acid bacteria can stimulate immunoglobulin A (IgA) production and modulate cytokine balance, promoting an anti-inflammatory immune tone [160,161]. As described earlier, bioactive peptides and exopolysaccharides formed during fermentation also exert immunomodulatory effects, enhancing resistance to pathogens, while vitamins synthesized microbially (e.g., folate, B_12_, and K_2_) support cellular immune processes. Human and experimental studies involving kefir consumption report increased natural killer (NK) cell activity and enhanced mucosal immunity, suggesting systemic immune engagement beyond the gut [162,163]. In addition, observational and laboratory studies link fermented vegetable intake with lower circulating inflammatory markers and improved immune balance [164,165,166,167]. While these findings highlight plausible mechanisms and biological effects, clinical evidence remains limited, and well-controlled trials using standardized, region-specific fermented products are needed to confirm causal effects

### 5.4. Metabolic Health

In Eastern European diets, fermented dairy, vegetable, and cereal products may contribute to metabolic homeostasis by influencing glucose regulation, lipid metabolism, and inflammatory balance. Through the combined action of fermentation-derived peptides, short-chain fatty acids, and transformed polyphenols, these foods are plausibly linked to improved insulin sensitivity, glucose handling, and attenuation of low-grade inflammation associated with metabolic disorders [168,169]. Fermentation of cereals, particularly in rye sourdough, reduces the glycemic index of bread and increases mineral bioavailability through phytate degradation and organic acid formation [73]. Intervention studies in adults with metabolic syndrome report modest reductions in fasting glucose and improvements in lipid profiles following regular consumption of kefir or fermented vegetables [170,171]. Similarly, rye sourdough bread elicits a lower postprandial glucose response than industrial yeast breads, likely due to organic acids and fermentation-induced peptide activity [77,172,173,174]. However, human evidence remains limited, and further controlled trials with traditional Eastern European products are warranted.

## 6. Comparative Perspective

Eastern European fermented foods occupy a distinct niche within global fermentation traditions, both in terms of microbial composition and functional properties. While many fermented foods share general mechanisms—such as lactic acid production, bioactive peptide generation, and polyphenol enhancement—regional variations create unique nutritional and functional signatures (Table 4).

### 6.1. Microbial Diversity

Eastern European fermentations often rely on spontaneous or mixed-culture fermentation, resulting in complex consortia of lactic acid bacteria (*Lactobacillus*, *Leuconostoc*, *Lactococcus*), acetic acid bacteria, and yeasts. In contrast, many Western commercial products (e.g., industrial yogurt) employ single-strain or defined starter cultures, which produce more consistent but less diverse microbial profiles [175]. Asian fermentations, such as kimchi or miso, include region-specific microbial species, but raw materials (e.g., soy, chili, seaweed) and flavor profiles differ substantially from the cabbage-, dairy-, and cereal-based Eastern European repertoire [30]. Metagenomic studies confirm that spontaneous fermentations typical of Eastern Europe harbor a higher microbial richness and functional gene diversity than controlled starter fermentations [176,177]. Such diversity enables cooperative and sequential metabolism among taxa, generating a broader range of bioactive peptides, organic acids, and volatile compounds. This microbial diversity contributes to distinctive flavor, aroma, and functional metabolite profiles, potentially supporting broader physiological effects.

### 6.2. Substrates and Nutrient Matrix

Vegetable substrates dominate in Eastern Europe (cabbage, beet, cucumbers), whereas fermented dairy is prominent in both Eastern Europe and Western Europe, and legumes/soy in Asia. In contrast to soy- and pulse-based Asian ferments or milk-centered Western ones, Eastern European traditions combine vegetables, cereals, and dairy—substrates that together yield a nutritionally dense, microbially active matrix well adapted to cold climates and seasonal food scarcity [178]. Cereal-based fermentations (rye sourdough, kvass) are particularly characteristic of Eastern Europe and northern climates, providing unique bioactive peptides and organic acids not commonly found in Asian or Western diets. In this context, seasonality and traditional preparation influence the nutrient matrix and bioactive composition of these foods.

### 6.3. Functional Implications

In this comparative context, the synergy of microbial diversity and substrate composition in Eastern European fermented foods results in distinct functional properties, including a broader spectrum of bioactive peptides, diverse postbiotic metabolites, and multiple micronutrient enhancements. While similar functional claims are made for Asian and Western fermented foods, the integration of cereals, vegetables, and dairy in Eastern Europe is relatively distinctive, offering a more complex combination of bioactives within single dietary items [147,178]. This integrative pattern may contribute to the functional resilience of traditional Eastern European diets, which combine plant-based ferments with dairy matrices, providing complementary microbial and metabolic stimuli that may support metabolic, immune, and gut health simultaneously.

### 6.4. Cultural Context and Persistence

The persistence of household-level fermentation practices in Eastern Europe, maintained across centuries, probably as a reaction to food scarcity but also as a result of own harvest preservation, supports ongoing exposure to diverse microbial and bioactive profiles. This continuity preserves both microbial and biochemical biodiversity that are increasingly eroded in industrialized food systems, linking cultural resilience with potential health resilience. Distinct regional varieties illustrate this diversity: Romanian borș (a wheat-bran ferment used as a soup base), Polish kapusta kiszona (traditional sauerkraut), Slovak bryndza (fermented sheep cheese), and Russian or Ukrainian kvass (mildly fermented cereal beverage) exemplify how fermentation adapted to local raw materials and seasonal cycles. These products, though simple in preparation, maintain complex microbial ecosystems that reflect centuries of empirical selection and household-level innovation, linking food preservation directly to identity and resilience. This contrasts with regions where industrial production dominates, potentially limiting microbial diversity and functional variation. Traditional knowledge, seasonal cycles, and communal practices ensure that functional potential is closely tied to cultural heritage, reinforcing both dietary and health impacts.

## 7. Challenges and Research Gaps

Despite the long-standing consumption and apparent functional potential of Eastern European fermented foods, several scientific and methodological challenges limit a comprehensive understanding of their health effects.

### 7.1. Heterogeneity of Traditional Practices

Preparation methods vary widely by region, household, and season, resulting in substantial variability in microbial composition, metabolite content, and nutrient profiles. Standardization for research purposes remains difficult, as most published studies rely on commercially produced or laboratory-fermented samples that may not accurately represent traditional products. This heterogeneity complicates reproducibility and cross-study comparison [10].

### 7.2. Limited Clinical Evidence

While in vitro and animal studies provide strong mechanistic support, well-designed human trials remain scarce, as explained previously in this article. Existing studies are typically small, short-term, or observational, limiting the ability to infer causality or establish dose–response relationships. Few clinical investigations directly compare traditional fermented foods with industrial analogs, leaving uncertainty about the magnitude of their specific health impacts.

### 7.3. Bioavailability and Mechanistic Understanding

The bioavailability of bioactive peptides, polyphenols, and vitamins in fermented foods remains insufficiently characterized. The presence of these compounds does not necessarily ensure efficient absorption or biological activity. Interactions between microbial metabolites and host physiology—including modulation of the gut microbiota, epithelial signaling, and metabolic pathways—are incompletely understood [147,179]. Longitudinal and multi-omic studies are needed to clarify how habitual consumption influences chronic disease outcomes.

### 7.4. Safety and Quality Considerations

Some traditional products contain high salt levels (notably fermented vegetables) or trace ethanol (in cereal-based beverages such as kvass), which may present health risks for sensitive populations [32,180,181,182]. This highlights the importance of hygienic practices, consumer education, and microbial monitoring to ensure both safety and functional integrity. In non-standardized household fermentations, inadequate hygiene, temperature control, or raw material quality may increase the risk of spoilage or, less frequently, pathogenic microbial contamination. In addition, certain fermented foods—particularly protein-rich matrices—may accumulate biogenic amines when fermentation is not adequately controlled. From a public health perspective, emerging strategies such as partial sodium replacement (e.g., potassium chloride), optimization of fermentation parameters, and the use of selected starter cultures may help reduce sodium content while preserving microbial safety and sensory quality

### 7.5. Integration with Modern Nutrition Science

Although traditional knowledge provides a valuable foundation, systematic scientific integration remains limited. Translating artisanal practices into evidence-based dietary recommendations or functional food innovations requires comprehensive nutritional, microbial, and metabolomic characterization. The application of omics technologies can help map microbial and metabolite diversity, link them to health endpoints, and guide the rational development of standardized, culturally grounded fermented foods [183]. Addressing these challenges will require interdisciplinary collaboration among microbiologists, nutritionists, food technologists, and public health experts. Harmonized methodologies, region-specific food characterization, and large-scale human intervention trials are critical steps toward validating the health-promoting potential of Eastern European fermented foods within modern nutrition science. Despite the deep-rooted presence of fermentation across Eastern Europe, scientific documentation and coordinated research remain uneven. Most published data originate from Poland, Romania, and the Czech Republic, while Balkan and Baltic countries are underrepresented in the international literature. Establishing regional collaborations or EU-supported consortia could facilitate standardized methods, microbial biobanking, and cross-country clinical research. Such initiatives would elevate the global visibility of Eastern European food heritage and accelerate its integration into evidence-based nutrition and functional food science.

## 8. Conclusions and Perspectives

Eastern European fermented foods represent a diverse group of culturally embedded products with meaningful nutritional, microbiological, and functional potential. Although many of these foods are prepared through long-standing traditional practices, scientific investigation has only partially explored their microbial ecology, bioactive compounds, and potential health effects. This review consolidates current knowledge and emphasizes that these products may contribute beneficial microorganisms, metabolites, and fermentation-derived bioactives that support digestive, metabolic, and immune functions. However, the strength of evidence varies considerably, with most findings derived from in vitro or animal studies, and only limited human data available. A clearer understanding of these foods is increasingly relevant for public health, given rising consumer interest in fermented products and the growing recognition of the microbiome’s role in health. To advance the field, future research should prioritize (i) comprehensive microbiological and metabolomic profiling of representative traditional and industrial products; (ii) standardized characterization of bioactive compounds and their effective concentrations; and (iii) well-designed clinical studies assessing specific health outcomes. Strengthening safety assessments—regarding salt content, biogenic amines, and fermentation hygiene—will also be essential for informed dietary recommendations. By integrating cultural knowledge with emerging scientific evidence, this review highlights the importance and untapped scientific value of Eastern European fermented foods. Further interdisciplinary work will support their responsible use in public health strategies and functional food innovation, while preserving the rich culinary heritage that shapes these distinctive products.

## Figures and Tables

**Table 1:** Identified bioactive peptides in traditional Eastern European fermented foods.

Food	Representative Peptide(s) or Class	Reported Bioactivity	Key Reference(s) Cited
Kefir	Tripeptides Val–Pro–Pro (VPP) and Ile–Pro–Pro (IPP); multiple casein-derived peptides (LC–MS identified)	ACE-inhibitory (antihypertensive), antioxidant, immunomodulatory (varies by peptide)	[54,55,56]
Bryndza (sheep-milk cheese)	Casein-derived peptides (including motifs similar to VPP/IPP reported in ripened cheeses); specific peptide sequencing in bryndza is limited	ACE-inhibitory, antioxidant (reported for ripened cheeses generally)	[57,58]
Rye sourdough	A range of small peptides (LC–MS identified) derived from rye storage proteins and glutenins	Antioxidant and modest ACE-inhibitory activities reported; contribute to improved digestibility and lower postprandial glycemia	[59,60,61]

Notes: Where specific peptide sequences in traditional Eastern European products are not reported in primary studies, the table indicates peptide classes or motifs documented in similar fermented dairy or cereal matrices. VPP = Val–Pro–Pro; IPP = Ile–Pro–Pro.

**Table 3:** Health effects of taxa in Eastern European fermented products.

Mechanism	Proposed Effects	Key References
Competitive exclusion of pathogens	LAB adheres to intestinal mucosa, inhibiting *E. coli*, *Salmonella*, *Listeria* spp. via bacteriocins and organic acids	[104,105]
Modulation of mucosal immunity	Increased secretion of IgA, IL-10; downregulation of proinflammatory cytokines	[106]
Enhancement of intestinal barrier	LAB upregulates tight-junction proteins (occludin, claudin) and short-chain fatty acid (SCFA) production	[107,108]
Influence on gut microbiota	Fermented foods increase microbial diversity and beneficial genera (e.g., *Bifidobacterium*, *Faecalibacterium*) in human trials	[105,109]

**Table 4:** Comparison between fermented foods worldwide.

Region	Common Substrates	Microbial Diversity	Nutritional Role	Environmental Advantages
**Eastern Europe**	Vegetables (e.g., sauerkraut), cereals (e.g., rye sourdough, kvass), dairy (e.g., kefir)	Mixed cultures: lactic acid bacteria (LAB), yeasts, acetic acid bacteria	Rich in fiber, bioactive peptides, organic acids, probiotics; supports gut health and immune tone	Adapted to cold climates and seasonal scarcity; preservation-focused
**Asia**	Soy (e.g., miso, tempeh), pulses, rice, vegetables	Spontaneous or starter-enhanced LAB, fungi (*Aspergillus*, *Rhizopus*), yeasts	High in plant protein, umami compounds, and microbial metabolites; supports digestion and metabolic health	Tropical to temperate climates; fermentation used for flavor, preservation, and protein enhancement
**Western (Industrial)**	Primarily dairy (e.g., yogurt, cheese	Defined starter cultures: single-strain LAB (e.g., *Lactobacillus delbrueckii*, *Streptococcus thermophilus*)	Consistent probiotic delivery, calcium, B vitamins; less microbial diversity	Refrigeration-based preservation; less seasonal dependence; focused on standardization

## Data Availability

No new data were created or analyzed in this study. Data sharing is not applicable to this article.

## References

[1] McGovern P.E., Zhang J., Tang J., Zhang Z., Hall G.R., Moreau R.A., Nuñez A., Butrym E.D., Richards M.P., Wang C. (2004). Fermented Beverages of Pre- and Proto-Historic China. Proc. Natl. Acad. Sci. USA.

[2] Şanlier N., Gökcen B.B., Sezgin A.C. (2019). Health Benefits of Fermented Foods. Crit. Rev. Food Sci. Nutr..

[3] Apalowo O.E., Adegoye G.A., Mbogori T., Kandiah J., Obuotor T.M. (2024). Nutritional Characteristics, Health Impact, and Applications of Kefir. Foods.

[4] Raak C., Ostermann T., Boehm K., Molsberger F. (2014). Regular Consumption of Sauerkraut and Its Effect on Human Health: A Bibliometric Analysis. Glob. Adv. Health Med..

[5] Kaszuba J., Jańczak-Pieniążek M., Migut D., Kapusta I., Buczek J. (2024). Comparison of the Antioxidant and Sensorial Properties of Kvass Produced from Mountain Rye Bread with the Addition of Selected Plant Raw Materials. Foods.

[6] Skowron K., Budzyńska A., Grudlewska-Buda K., Wiktorczyk-Kapischke N., Andrzejewska M., Wałecka-Zacharska E., Gospodarek-Komkowska E. (2022). Two Faces of Fermented Foods—The Benefits and Threats of Its Consumption. Front. Microbiol..

[7] Food Consumption Data|EFSA. https://www.efsa.europa.eu/en/data-report/food-consumption-data.

[8] The Global Tradition of Fermented Foods: A Culinary Staple Across Cultures. https://www.thefermented.org/learn/the-global-tradition-of-fermented-foods-a-culinary-staple-across-cultures.

[9] Wiśniewska M.Z., Kowalska A., Manning L., Malinowska E., Kowalski P. (2022). Consumption and Home Preparation of Fermented Vegetable Products in Poland. Zesz. Nauk. Organ. Zarządzanie Politech. Śląska.

[10] Sõukand R., Pieroni A., Biró M., Dénes A., Dogan Y., Hajdari A., Kalle R., Reade B., Mustafa B., Nedelcheva A. (2015). An Ethnobotanical Perspective on Traditional Fermented Plant Foods and Beverages in Eastern Europe. J. Ethnopharmacol..

[11] Steinkraus K.H. (1997). Classification of Fermented Foods: Worldwide Review of Household Fermentation Techniques. Food Control.

[12] Tamang J.P., Watanabe K., Holzapfel W.H. (2016). Review: Diversity of Microorganisms in Global Fermented Foods and Beverages. Front. Microbiol..

[13] Sauerkraut History and Use in Eastern European Recipes. https://web.archive.org/web/20170116055455/http://easteuropeanfood.about.com/od/vegetables/a/sauerkraut.htm.

[14] Horvath S., Vass E., Ardelean E. (2017). Alimentaţia Tradiţională din Bihor Între Secolul al XVIII-Lea şi Începutul Secolului al XX-Lea.

[15] Beet Kvass: An Unbeetable Traditional Ukrainian Health Drink|Tin and Thyme. https://tinandthyme.uk/2018/05/beet-kvass/.

[16] Netmeds|www.netmeds.com. https://www.netmeds.com/c/health-library/post/sauerkraut-nutrition-uses-health-benefits-and-know-how-to-make-this-probiotic-food-at-home.

[17] Knight S., Klaere S., Fedrizzi B., Goddard M.R. (2015). Regional Microbial Signatures Positively Correlate with Differential Wine Phenotypes: Evidence for a Microbial Aspect to Terroir. Sci. Rep..

[18] Prado M.R., Blandón L.M., Vandenberghe L.P.S., Rodrigues C., Castro G.R., Thomaz-Soccol V., Soccol C.R. (2015). Milk Kefir: Composition, Microbial Cultures, Biological Activities, and Related Products. Front. Microbiol..

[19] Ряженка: Пoльза, Прoизвoдствo и Рецепты Этoгo Традициoннoгo Кислoмoлoч. https://gastronomusa.com/ru/blogs/blogs/ryazhenka-benefits-production-and-recipes-with-this-traditional-dairy-drink.

[20] Altay F., Shah N.P. (2017). Chapter 17—Rheology and Functionality of Ayran—A Yogurt Drink. Yogurt in Health and Disease Prevention.

[21] Teneva-Angelova T., Balabanova T., Boyanova P., Beshkova D. (2018). Traditional Balkan Fermented Milk Products. Eng. Life Sci..

[22] Metchnikoff É., Chalmers Mitchell P. (1908). The Prolongation of Life: Optimistic Studies.

[23] 4 Best Dairy Products in Eastern Europe—TasteAtlas. https://www.tasteatlas.com/best-rated-dairy-products-in-eastern-europe.

[24] Maciuc V., Pânzaru C., Ciocan-Alupii M., Radu-Rusu C.-G., Radu-Rusu R.-M. (2024). Comparative Assessment of the Nutritional and Sanogenic Features of Certain Cheese Sorts Originating in Conventional Dairy Farms and in “Mountainous” Quality System Farms. Agriculture.

[25] Tsafrakidou P., Michaelidou A.M., Biliaderis C.G. (2020). Fermented Cereal-Based Products: Nutritional Aspects, Possible Impact on Gut Microbiota and Health Implications. Foods.

[26] Iversen K.N., Dicksved J., Zoki C., Fristedt R., Pelve E.A., Langton M., Landberg R. (2022). The Effects of High Fiber Rye, Compared to Refined Wheat, on Gut Microbiota Composition, Plasma Short Chain Fatty Acids, and Implications for Weight Loss and Metabolic Risk Factors (the RyeWeight Study). Nutrients.

[27] blog V.-B. ’n B. Ancient Russian Fermented Kissel Porridg|Beets & Bones 2018. https://www.beetsandbones.com/russian-fermented-kissel-porridge/.

[28] Puskar L. Why Eastern Europeans Revere Black Bread. https://www.greenseashells.com/post/why-eastern-europeans-revere-black-bread.

[29] 15 Best Sourdough Breads in Europe. https://www.tasteatlas.com/best-rated-sourdough-breads-in-europe.

[30] Lee C.-H., Ahn J., Son H.-S. (2024). Ethnic Fermented Foods of the World: An Overview. J. Ethn. Foods.

[31] Ignatenko B.V., Nikolaeva M.A., Eliseev M.N. (2021). History of kvass as a national drink. Tovaroved Prodovol. Tovarov Commod. Spec. Food Prod..

[32] Dlusskaya E., Jänsch A., Schwab C., Gänzle M.G. (2008). Microbial and Chemical Analysis of a Kvass Fermentation. Eur. Food Res. Technol..

[33] This Ancient East European Brew Repurposes Leftover Bread—To Great Effect. https://www.sbs.com.au/food/article/kvass-fermented-drink/gwnxipi2f.

[34] Borș Wheat Bran Fermented Beverage—Feastern Europe—Recipe. Feastern Europe 2021. https://feasterneurope.com/bors-recipe/.

[35] Pasqualone A., Summo C., Laddomada B., Mudura E., Coldea T.E. (2018). Effect of Processing Variables on the Physico-Chemical Characteristics and Aroma of *Borş*, a Traditional Beverage Derived from Wheat Bran. Food Chem..

[36] Chandrasekaran R., Shanmugham B., Tiwari A., Sarma H. (2025). Cereal-Based Ethnic Fermented Food Products and Low Alcoholic Beverages: Preparation Methods, Nutritional Quality, and Their Health Benefits. Ethnic and Indigenous Food Technologies: Role in Climate and Health Resilience.

[37] Ashaolu T.J., Varga L., Greff B. (2025). Nutritional and Functional Aspects of European Cereal-Based Fermented Foods and Beverages. Food Res. Int..

[38] Wang B., Rutherfurd-Markwick K., Zhang X.-X., Mutukumira A.N. (2022). Kombucha: Production and Microbiological Research. Foods.

[39] Bishop P., Pitts E.R., Budner D., Thompson-Witrick K.A. (2022). Chemical Composition of Kombucha. Beverages.

[40] Andrade D.K.A., Wang B., Lima E.M.F., Shebeko S.K., Ermakov A.M., Khramova V.N., Ivanova I.V., Rocha R.d.S., Vaz-Velho M., Mutukumira A.N. (2025). Kombucha: An Old Tradition into a New Concept of a Beneficial, Health-Promoting Beverage. Foods.

[41] Samtiya M., Aluko R.E., Puniya A.K., Dhewa T. (2021). Enhancing Micronutrients Bioavailability through Fermentation of Plant-Based Foods: A Concise Review. Fermentation.

[42] Knez E., Kadac-Czapska K., Grembecka M. (2023). Effect of Fermentation on the Nutritional Quality of the Selected Vegetables and Legumes and Their Health Effects. Life.

[43] Sarmale, a Symbol of the Delicious Mix Romanian Gastronomy Has to Offer|RBI Insights. https://www.rbinternational.com/en/raiffeisen/blog/events-lifestyle/sarmale-romanian-gastronomy.html.

[44] Transylvania Unveiled. https://www.transylvaniaunveiled.com/blog/sarmale.

[45] Sarmale de Post (Vegetarian Stuffed Cabbage), Radio Romania International. https://www.rri.ro/rubrici/secretele-bucatariei-romanesti/sarmale-de-post-3-id594208.html.

[46] History of Traditional Russian Drink Kvass and Cultural Significance—How To Russia. https://howtorussia.com/.

[47] The History of Kefir: A Journey Through Cultures, Mountains, and Mysteries. https://kefir.tw/en/the-history-of-kefir-a-journey-through-cultures-mountains-and-mysteries/.

[48] Pickling and Preserving in Bulgarian Food Culture|Copernico. Geschichte Und Kulturelles Erbe Im Östlichen Europa. https://www.copernico.eu/en/articles/pickling-and-preserving-bulgarian-food-culture.

[49] 80% Dintre Romani Conserva Muraturi in Casa|Progresiv. https://revistaprogresiv.ro/arhive/80-dintre-romani-conserva-muraturi-casa/.

[50] Fitsum S., Gebreyohannes G., Sbhatu D.B. (2025). Bioactive Compounds in Fermented Foods: Health Benefits, Safety, and Future Perspectives. Appl. Food Res..

[51] Park I., Mannaa M. (2025). Fermented Foods as Functional Systems: Microbial Communities and Metabolites Influencing Gut Health and Systemic Outcomes. Foods.

[52] Natural Ingredient Trends. Global Market Overview. Top 10 Trends. https://www.innovamarketinsights.com/trends/natural-ingredient-trends/.

[53] Zaky A.A., Simal-Gandara J., Eun J.-B., Shim J.-H., Abd El-Aty A.M. (2021). Bioactivities, Applications, Safety, and Health Benefits of Bioactive Peptides from Food and By-Products: A Review. Front. Nutr..

[54] Ebner J., Aşçı Arslan A., Fedorova M., Hoffmann R., Küçükçetin A., Pischetsrieder M. (2015). Peptide Profiling of Bovine Kefir Reveals 236 Unique Peptides Released from Caseins during Its Production by Starter Culture or Kefir Grains. J. Proteom..

[55] Quirós A., Hernández-Ledesma B., Ramos M., Amigo L., Recio I. (2005). Angiotensin-Converting Enzyme Inhibitory Activity of Peptides Derived from Caprine Kefir. J. Dairy Sci..

[56] Tagliazucchi D., Martini S., Solieri L. (2019). Bioprospecting for Bioactive Peptide Production by Lactic Acid Bacteria Isolated from Fermented Dairy Food. Fermentation.

[57] Jäkälä P., Vapaatalo H. (2010). Antihypertensive Peptides from Milk Proteins. Pharmaceuticals.

[58] Helal A., Tagliazucchi D. (2023). Peptidomics Profile, Bioactive Peptides Identification and Biological Activities of Six Different Cheese Varieties. Biology.

[59] Koistinen V.M., Mattila O., Katina K., Poutanen K., Aura A.-M., Hanhineva K. (2018). Metabolic Profiling of Sourdough Fermented Wheat and Rye Bread. Sci. Rep..

[60] Katina K., Arendt E., Liukkonen K.-H., Autio K., Flander L., Poutanen K. (2005). Potential of Sourdough for Healthier Cereal Products. Trends Food Sci. Technol..

[61] Poutanen K., Flander L., Katina K. (2009). Sourdough and Cereal Fermentation in a Nutritional Perspective. Food Microbiol..

[62] Şahingil D., Atalay M. (2022). Characterization of Antimicrobial Peptide Fraction Producing *Lactobacillus* Spp. Based on LC/MS-MS and Determination of ACE-Inhibitory Activity in Kefir. J. Agric. Sci..

[63] Hirota T., Ohki K., Kawagishi R., Kajimoto Y., Mizuno S., Nakamura Y., Kitakaze M. (2007). Casein Hydrolysate Containing the Antihypertensive Tripeptides Val-Pro-Pro and Ile-Pro-Pro Improves Vascular Endothelial Function Independent of Blood Pressure-Lowering Effects: Contribution of the Inhibitory Action of Angiotensin-Converting Enzyme. Hypertens. Res..

[64] Dalabasmaz S., de la Torre E.P., Gensberger-Reigl S., Pischetsrieder M., Rodríguez-Ortega M.J. (2023). Identification of Potential Bioactive Peptides in Sheep Milk Kefir through Peptidomic Analysis at Different Fermentation Times. Foods.

[65] Pihlanto A. (2013). Lactic Fermentation and Bioactive Peptides. Lactic Acid Bacteria—R & D for Food, Health and Livestock Purposes.

[66] de Oliveira Leite A.M., Miguel M.A., Peixoto R.S., Rosado A.S., Silva J.T., Paschoalin V.M. (2013). Microbiological, technological and therapeutic properties of kefir: A natural probiotic beverage. Braz J Microbiol..

[67] Skulska I.V., Tsisaryk O.Y. (2020). Changes in Protein Substances of Brynza Cheese under the Influence of Partial Replacement of Salt with Potassium Chloride. Sci. Messenger LNU Vet. Med. Biotechnol. Ser. Food Technol..

[68] Kačániová M., Terentjeva M., Kunová S., Haščík P., Kowalczewski P.Ł., Štefániková J. (2021). Diversity of Microbiota in Slovak Summer Ewes’ Cheese “Bryndza”. Open Life Sci..

[69] Kačániová M., Nagyová Ľ., Štefániková J., Felsöciová S., Godočí-ková L., Haščí-k P., Horská E., Kunová S. (2020). The Characteristic of Sheep Cheese “Bryndza” from Different Regions of Slovakia Based on Microbiological Quality. Potravin. Slovak J. Food Sci..

[70] Manso M.A., López-Fandiño R. (2003). Angiotensin I Converting Enzyme-Inhibitory Activity of Bovine, Ovine, and Caprine Kappa-Casein Macropeptides and Their Tryptic Hydrolysates. J. Food Prot..

[71] Mohanty D., Jena R., Choudhury P.K., Pattnaik R., Mohapatra S., Saini M.R. (2016). Milk Derived Antimicrobial Bioactive Peptides: A Review. Int. J. Food Prop..

[72] Contreras M.d.M., Carrón R., Montero M.J., Ramos M., Recio I. (2009). Novel Casein-Derived Peptides with Antihypertensive Activity. Int. Dairy J..

[73] Bhandari D., Rafiq S., Gat Y., Gat P., Waghmare R., Kumar V. (2020). A Review on Bioactive Peptides: Physiological Functions, Bioavailability and Safety. Int. J. Pept. Res. Ther..

[74] Coda R., Rizzello C.G., Pinto D., Gobbetti M. (2012). Selected Lactic Acid Bacteria Synthesize Antioxidant Peptides during Sourdough Fermentation of Cereal Flours. Appl. Environ. Microbiol..

[75] Maioli M., Pes G.M., Sanna M., Cherchi S., Dettori M., Manca E., Farris G.A. (2008). Sourdough-Leavened Bread Improves Postprandial Glucose and Insulin Plasma Levels in Subjects with Impaired Glucose Tolerance. Acta Diabetol..

[76] Iversen K.N., Jonsson K., Landberg R. (2022). The Effect of Rye-Based Foods on Postprandial Plasma Insulin Concentration: The Rye Factor. Front. Nutr..

[77] Reduction of Post-Prandial Glycaemic Responses Compared with Glucose|EFSA. https://www.efsa.europa.eu/en/efsajournal/pub/3837.

[78] Ribet L., Dessalles R., Lesens C., Brusselaers N., Durand-Dubief M. (2022). Nutritional Benefits of Sourdoughs: A Systematic Review. Adv. Nutr..

[79] Abbaspour N. (2024). Fermentation’s Pivotal Role in Shaping the Future of Plant-Based Foods: An Integrative Review of Fermentation Processes and Their Impact on Sensory and Health Benefits. Appl. Food Res..

[80] Chaudhary A., Bhalla S., Patiyal S., Raghava G.P.S., Sahni G. (2021). FermFooDb: A Database of Bioactive Peptides Derived from Fermented Foods. Heliyon.

[81] Wang Y., Zhang Y., Li S., Huang K., Cao H., Qiu W., Guan X. (2025). Mechanism of the Release and Transformation of Polyphenols during Germination and Fermentation in Millets: Profile and Metabolomics Based Analysis. Food Funct..

[82] Oluwole O., Fernando W.B., Lumanlan J., Ademuyiwa O., Jayasena V. (2022). Role of Phenolic Acid, Tannins, Stilbenes, Lignans and Flavonoids in Human Health—A Review. Int. J. Food Sci. Technol..

[83] Intharuksa A., Kuljarusnont S., Sasaki Y., Tungmunnithum D. (2024). Flavonoids and Other Polyphenols: Bioactive Molecules from Traditional Medicine Recipes/Medicinal Plants and Their Potential for Phytopharmaceutical and Medical Application. Molecules.

[84] Yang F., Chen C., Ni D., Yang Y., Tian J., Li Y., Chen S., Ye X., Wang L. (2023). Effects of Fermentation on Bioactivity and the Composition of Polyphenols Contained in Polyphenol-Rich Foods: A Review. Foods.

[85] Ripari V., Bai Y., Gänzle M.G. (2019). Metabolism of Phenolic Acids in Whole Wheat and Rye Malt Sourdoughs. Food Microbiol..

[86] Miyagusuku-Cruzado G., García-Cano I., Rocha-Mendoza D., Jiménez-Flores R., Giusti M.M. (2020). Monitoring Hydroxycinnamic Acid Decarboxylation by Lactic Acid Bacteria Using High-Throughput UV-Vis Spectroscopy. Molecules.

[87] López de Felipe F. (2023). Revised Aspects into the Molecular Bases of Hydroxycinnamic Acid Metabolism in Lactobacilli. Antioxidants.

[88] Hu H., Li L., Ding S. (2015). An Organic Solvent-Tolerant Phenolic Acid Decarboxylase from Bacillus Licheniformis for the Efficient Bioconversion of Hydroxycinnamic Acids to Vinyl Phenol Derivatives. Appl. Microbiol. Biotechnol..

[89] Dissanayake I.H., Tabassum W., Alsherbiny M., Chang D., Li C.G., Bhuyan D.J. (2025). Lactic Acid Bacterial Fermentation as a Biotransformation Strategy to Enhance the Bioavailability of Phenolic Antioxidants in Fruits and Vegetables: A Comprehensive Review. Food Res. Int..

[90] Hsieh C.-C., Liu Y.-H., Lin S.-P., Santoso S.P., Jantama K., Tsai T.-Y., Hsieh C.-W., Cheng K.-C. (2024). Development of High-Glucosinolate-Retaining Lactic-Acid-Bacteria-Co-Fermented Cabbage Products. Fermentation.

[91] Mulțescu M., Susman I.-E., Culețu A., Zamfir M., Sanmartin A.M., Israel-Roming F. (2024). The influence of starter cultures of lactic acid bacteria on fermentation of white cabbage. AgroLife Sci. J..

[92] Wei L., Marco M.L. (2025). The Fermented Cabbage Metabolome and Its Protection against Cytokine-Induced Intestinal Barrier Disruption of Caco-2 Monolayers. Appl. Environ. Microbiol..

[93] Šalić A., Šamec D. (2022). Changes in the Content of Glucosinolates, Polyphenols and Carotenoids during Lactic-Acid Fermentation of Cruciferous Vegetables: A Mini Review. Food Chem. X.

[94] Rehman S., Mufti I.U., Ain Q.U., Ijaz B. (2024). Bioactive Compounds and Biological Activities of Red Beetroot (*Beta vulgaris* L.). Bioactive Compounds in the Storage Organs of Plants.

[95] Jakubczyk K., Melkis K., Janda-Milczarek K., Skonieczna-Żydecka K. (2024). Phenolic Compounds and Antioxidant Properties of Fermented Beetroot Juices Enriched with Different Additives. Foods.

[96] Duyar S.M., Sarı F., Karaoglan H.A. (2024). Degradation Kinetics of Betalains in Red Beetroot Juices throughout Fermentation Process and Storage. Ital. J. Food Sci..

[97] Chou Y.-C., Lin H.-W., Wang C.-Y., Hsieh C.-C., Santoso S.P., Lin S.-P., Cheng K.-C. (2024). Enhancing Antioxidant Benefits of Kombucha Through Optimized Glucuronic Acid by Selected Symbiotic Fermentation Culture. Antioxidants.

[98] Koistinen V.M. (2019). Effects of Food Processing and Gut Microbial Metabolism on Whole Grain Phytochemicals: A Metabolomics Approach.

[99] Alkay Z., Falah F., Cankurt H., Dertli E. (2024). Exploring the Nutritional Impact of Sourdough Fermentation: Its Mechanisms and Functional Potential. Foods.

[100] Saimaiti A., Huang S.-Y., Xiong R.-G., Wu S.-X., Zhou D.-D., Yang Z.-J., Luo M., Gan R.-Y., Li H.-B. (2022). Antioxidant Capacities and Polyphenol Contents of Kombucha Beverages Based on Vine Tea and Sweet Tea. Antioxidants.

[101] de Oliveira P.V., da Silva Júnior A.H., de Oliveira C.R.S., Assumpção C.F., Ogeda C.H. (2023). Kombucha Benefits, Risks and Regulatory Frameworks: A Review. Food Chem. Adv..

[102] Kiczorowski P., Kiczorowska B., Samolińska W., Szmigielski M., Winiarska-Mieczan A. (2022). Effect of Fermentation of Chosen Vegetables on the Nutrient, Mineral, and Biocomponent Profile in Human and Animal Nutrition. Sci. Rep..

[103] Tlais A.Z.A., Lemos Junior W.J.F., Filannino P., Campanaro S., Gobbetti M., Di Cagno R. (2022). How Microbiome Composition Correlates with Biochemical Changes during Sauerkraut Fermentation: A Focus on Neglected Bacterial Players and Functionalities. Microbiol. Spectr..

[104] Garofalo C., Osimani A., Milanović V., Aquilanti L., De Filippis F., Stellato G., Di Mauro S., Turchetti B., Buzzini P., Ercolini D. (2015). Bacteria and Yeast Microbiota in Milk Kefir Grains from Different Italian Regions. Food Microbiol..

[105] Wastyk H.C., Fragiadakis G.K., Perelman D., Dahan D., Merrill B.D., Yu F.B., Topf M., Gonzalez C.G., Treuren W.V., Han S. (2021). Gut-Microbiota-Targeted Diets Modulate Human Immune Status. Cell.

[106] de Moreno de LeBlanc A., del Carmen S., Zurita-Turk M., Santos Rocha C., van de Guchte M., Azevedo V., Miyoshi A., LeBlanc J.G. (2011). Importance of IL-10 Modulation by Probiotic Microorganisms in Gastrointestinal Inflammatory Diseases. Int. Sch. Res. Not..

[107] Feng Y., Wang Y., Wang P., Huang Y., Wang F. (2018). Short-Chain Fatty Acids Manifest Stimulative and Protective Effects on Intestinal Barrier Function Through the Inhibition of NLRP3 Inflammasome and Autophagy. Cell. Physiol. Biochem..

[108] Gou H.-Z., Zhang Y.-L., Ren L.-F., Li Z.-J., Zhang L. (2022). How Do Intestinal Probiotics Restore the Intestinal Barrier?. Front. Microbiol..

[109] Marco M.L., Sanders M.E., Gänzle M., Arrieta M.C., Cotter P.D., De Vuyst L., Hill C., Holzapfel W., Lebeer S., Merenstein D. (2021). The International Scientific Association for Probiotics and Prebiotics (ISAPP) Consensus Statement on Fermented Foods. Nat. Rev. Gastroenterol. Hepatol..

[110] Davani-Davari D., Negahdaripour M., Karimzadeh I., Seifan M., Mohkam M., Masoumi S.J., Berenjian A., Ghasemi Y. (2019). Prebiotics: Definition, Types, Sources, Mechanisms, and Clinical Applications. Foods.

[111] Koj K., Pejcz E. (2023). Rye Dietary Fiber Components upon the Influence of Fermentation Inoculated with Probiotic Microorganisms. Molecules.

[112] Williams B.A., Grant L.J., Gidley M.J., Mikkelsen D. (2017). Gut Fermentation of Dietary Fibres: Physico-Chemistry of Plant Cell Walls and Implications for Health. Int. J. Mol. Sci..

[113] Dahiya D., Nigam P.S. (2023). Therapeutic and Dietary Support for Gastrointestinal Tract Using Kefir as a Nutraceutical Beverage: Dairy-Milk-Based or Plant-Sourced Kefir Probiotic Products for Vegan and Lactose-Intolerant Populations. Fermentation.

[114] Negishi H., Ichikawa A., Takahashi S., Kano H., Makino S. (2025). Targeted Prebiotic Application of Gluconic Acid-Containing Oligosaccharides Promotes Faecalibacterium Growth through Microbial Cross-Feeding Networks. ISME J..

[115] Du Y., He C., An Y., Huang Y., Zhang H., Fu W., Wang M., Shan Z., Xie J., Yang Y. (2024). The Role of Short Chain Fatty Acids in Inflammation and Body Health. Int. J. Mol. Sci..

[116] Collins S.M., Gibson G.R., Kennedy O.B., Walton G., Rowland I., Commane D.M. (2021). Development of a Prebiotic Blend to Influence in Vitro Fermentation Effects, with a Focus on Propionate, in the Gut. FEMS Microbiol. Ecol..

[117] Fehlbaum S., Prudence K., Kieboom J., Heerikhuisen M., Van den Broek T., Schuren F.H.J., Steinert R.E., Raederstorff D. (2018). In Vitro Fermentation of Selected Prebiotics and Their Effects on the Composition and Activity of the Adult Gut Microbiota. Int. J. Mol. Sci..

[118] Christensen C.M., Kok C.R., Auchtung J.M., Hutkins R. (2022). Prebiotics Enhance Persistence of Fermented-Food Associated Bacteria in in Vitro Cultivated Fecal Microbial Communities. Front. Microbiol..

[119] Whelan K., Alexander M., Gaiani C., Lunken G., Holmes A., Staudacher H.M., Theis S., Marco M.L. (2024). Design and Reporting of Prebiotic and Probiotic Clinical Trials in the Context of Diet and the Gut Microbiome. Nat. Microbiol..

[120] Gurunathan S., Thangaraj P., Kim J.-H. (2024). Postbiotics: Functional Food Materials and Therapeutic Agents for Cancer, Diabetes, and Inflammatory Diseases. Foods.

[121] Salek R.N., Pleva P., Sumczynski D., Vinter Š., Kopečková J., Rejdlová A., Lorencová E. (2025). Sauerkraut Juice Fermented with Different Symbiotic Starter Cultures: Comprehensive Assessment of Physicochemical, Rheological, Antioxidant, and Microbiological Characteristics. Front. Sustain. Food Syst..

[122] Jácome-Silva L.G., Marín-Iniesta F., Tortosa-Díaz L., Planes-Muñoz D., López-Nicolas R. (2025). Influence of the Type of Sauerkraut Fermentation with Probiotics Strains on Folate Content, Antioxidant Activity and Sensory Analysis. Appl. Sci..

[123] Bauer M. (2018). Sauerkraut Fermentation: Process, Microbiology, Defects and Spoilage|Industrial Microbiology. Biol. Discuss..

[124] Jastrząb R., Graczyk D., Siedlecki P. (2021). Molecular and Cellular Mechanisms Influenced by Postbiotics. Int. J. Mol. Sci..

[125] Aguilar-Toalá J.E., Garcia-Varela R., Garcia H.S., Mata-Haro V., González-Córdova A.F., Vallejo-Cordoba B., Hernández-Mendoza A. (2018). Postbiotics: An Evolving Term within the Functional Foods Field. Trends Food Sci. Technol..

[126] Nasreen S., Ali S., Andleeb S., Summer M., Hussain T., Imdad K., Ara C., Tahir H.M. (2024). Mechanisms of Medicinal, Pharmaceutical, and Immunomodulatory Action of Probiotics Bacteria and Their Secondary Metabolites against Disease Management: An Overview. Folia Microbiol..

[127] Wegh C.A.M., Geerlings S.Y., Knol J., Roeselers G., Belzer C. (2019). Postbiotics and Their Potential Applications in Early Life Nutrition and Beyond. Int. J. Mol. Sci..

[128] Schwarzer M., Tlaskalova-Hogenova H., Leulier F., Schabussova I. (2021). Editorial: Employing Experimental Gnotobiotic Models to Decipher the Host-Microbiota Cross-Talk in Health and Disease. Front. Immunol..

[129] Mukherjee A., Farsi D.N., Garcia-Gutierrez E., Akan E., Millan J.A.S., Angelovski L., Bintsis T., Gérard A., Güley Z., Kabakcı S. (2025). Impact of Fermented Foods Consumption on Gastrointestinal Wellbeing in Healthy Adults: A Systematic Review and Meta-Analysis. Front. Nutr..

[130] Chan M., Baxter H., Larsen N., Jespersen L., Ekinci E.I., Howell K. (2019). Impact of Botanical Fermented Foods on Metabolic Biomarkers and Gut Microbiota in Adults with Metabolic Syndrome and Type 2 Diabetes: A Systematic Review Protocol. BMJ Open.

[131] Gill P., Staudacher H.M. (2023). Are Postbiotics Key to the Potential Benefits of Fermented Foods?. Lancet Gastroenterol. Hepatol..

[132] Zdybel K., Śliwka A., Polak-Berecka M., Polak P., Waśko A. (2025). Postbiotics Formulation and Therapeutic Effect in Inflammation: A Systematic Review. Nutrients.

[133] Zhang K., Liu S., Liang S., Xiang F., Wang X., Lian H., Li B., Liu F. (2024). Exopolysaccharides of Lactic Acid Bacteria: Structure, Biological Activity, Structure-Activity Relationship, and Application in the Food Industry: A Review. Int. J. Biol. Macromol..

[134] Abitha Eswari U., Radhamanalan G., Dharumadurai D., Dharumadurai D. (2024). Thermal Methods of Postbiotics Preparation. Postbiotics.

[135] Da M., Sun J., Ma C., Li D., Dong L., Wang L.-S., Chen F. (2024). Postbiotics: Enhancing Human Health with a Novel Concept. eFood.

[136] LeBlanc J.G., Laiño J.E., del Valle M.J., Vannini V., van Sinderen D., Taranto M.P., de Valdez G.F., de Giori G.S., Sesma F. (2011). B-Group Vitamin Production by Lactic Acid Bacteria—Current Knowledge and Potential Applications. J. Appl. Microbiol..

[137] Hugenschmidt S., Schwenninger S.M., Lacroix C. (2011). Concurrent High Production of Natural Folate and Vitamin B12 Using a Co-Culture Process with *Lactobacillus plantarum* SM39 and *Propionibacterium freudenreichii* DF13. Process Biochem..

[138] Deptula P., Chamlagain B., Edelmann M., Sangsuwan P., Nyman T.A., Savijoki K., Piironen V., Varmanen P. (2017). Food-Like Growth Conditions Support Production of Active Vitamin B12 by Propionibacterium Freudenreichii 2067 without DMBI, the Lower Ligand Base, or Cobalt Supplementation. Front. Microbiol..

[139] Reale A., Konietzny U., Coppola R., Sorrentino E., Greiner R. (2007). The Importance of Lactic Acid Bacteria for Phytate Degradation during Cereal Dough Fermentation. J. Agric. Food Chem..

[140] Zhou D., Zhao Y., Li J., Ravichandran V., Wang L., Huang Q., Chen C., Ni H., Yin J. (2021). Effects of Phytic Acid-Degrading Bacteria on Mineral Element Content in Mice. Front. Microbiol..

[141] Molina G.E.S., Ras G., da Silva D.F., Duedahl-Olesen L., Hansen E.B., Bang-Berthelsen C.H. (2025). Metabolic Insights of Lactic Acid Bacteria in Reducing Off-Flavors and Antinutrients in Plant-Based Fermented Dairy Alternatives. Compr. Rev. Food Sci. Food Saf..

[142] Keyvan E., Adesemoye E., Champomier-Vergès M.-C., Chanséaume-Bussiere E., Mardon J., Nikolovska Nedelkoska D., Palamutoglu R., Russo P., Sarand I., Songre-Ouattara L. (2025). Vitamins Formed by Microorganisms in Fermented Foods: Effects on Human Vitamin Status—A Systematic Narrative Review. Front. Nutr..

[143] Sorathiya K.B., Melo A., Hogg M.C., Pintado M. (2025). Organic Acids in Food Preservation: Exploring Synergies, Molecular Insights, and Sustainable Applications. Sustainability.

[144] Gan J., Kong X., Wang K., Chen Y., Du M., Xu B., Xu J., Wang Z., Cheng Y., Yu T. (2023). Effect of Fermentation Using Different Lactic Acid Bacteria Strains on the Nutrient Components and Mineral Bioavailability of Soybean Yogurt Alternative. Front. Nutr..

[145] Gasaly N., Hermoso M.A., Gotteland M. (2021). Butyrate and the Fine-Tuning of Colonic Homeostasis: Implication for Inflammatory Bowel Diseases. Int. J. Mol. Sci..

[146] Xiao S., Jiang S., Qian D., Duan J. (2020). Modulation of Microbially Derived Short-Chain Fatty Acids on Intestinal Homeostasis, Metabolism, and Neuropsychiatric Disorder. Appl. Microbiol. Biotechnol..

[147] Künili İ.E., Akdeniz V., Akpınar A., Budak Ş.Ö., Curiel J.A., Guzel M., Karagözlü C., Berkel Kasikci M., Caruana G.P.M., Starowicz M. (2025). Bioactive Compounds in Fermented Foods: A Systematic Narrative Review. Front. Nutr..

[148] de Carvalho A.P.A., Conte-Junior C.A. (2024). Health and Bioactive Compounds of Fermented Foods and By-Products. Fermentation.

[149] Zhang J., Cianciosi D., Islam M.O., Zhang L. (2024). Editorial: Intervention Effects of Food-Derived Polyphenols and Bioactive Peptides on Chronic Inflammation. Front. Nutr..

[150] Iorizzo M., Di Martino C., Letizia F., Crawford T.W., Paventi G. (2024). Production of Conjugated Linoleic Acid (CLA) by Lactiplantibacillus Plantarum: A Review with Emphasis on Fermented Foods. Foods.

[151] Naseeb J., Kanwal M., Aldalali S., Sarwar A., Jamal S.B., Yang Z., Aziz T., Alharbi M., Shami A., Al-Asmari F. (2025). Metabolic and Enzymatic Characterization of Linoleic Acid Biotransformation by *Lactiplantibacillus plantarum* NGML2 to Conjugated Linoleic Acid and Different Metabolites. World J. Microbiol. Biotechnol..

[152] Rashidbeygi E., Samarin M.M., Sheikhhossein F., Khalilkhaneh A.H., Gholizadeh M., Lohrasbi N., Abbasi A., Bazyar H., Askari G., Amini M.R. (2025). The Effect of Kefir Consumption on Blood Pressure and C-Reactive Protein: A Systematic Review and Meta-Analysis of Randomised Controlled Trials. Endocrinol. Diabetes Metab..

[153] Tagliazucchi D., Martini S., Shamsia S., Helal A., Conte A. (2018). Biological Activities and Peptidomic Profile of in Vitro-Digested Cow, Camel, Goat and Sheep Milk. Int. Dairy J..

[154] Dimidi E., Cox S.R., Rossi M., Whelan K. (2019). Fermented Foods: Definitions and Characteristics, Impact on the Gut Microbiota and Effects on Gastrointestinal Health and Disease. Nutrients.

[155] Kefir’s Laxative Effects: Friend Or Foe?|MedShun. https://medshun.com/article/does-kefir-have-laxative-properties.

[156] Ki J.-H., Jung K.-O., Lee H.-S., Hwang I.-K., Kim Y.-J., Park K.-Y. (2004). Effects of Kimchi on Stomach and Colon Health of Helicobacter pylori—Infected Volunteers|DBpia. Prev. Nutr. Food Sci..

[157] Gao Y., Liu Y., Ma T., Liang Q., Sun J., Wu X., Song Y., Nie H., Huang J., Mu G. (2025). Fermented Dairy Products as Precision Modulators of Gut Microbiota and Host Health: Mechanistic Insights, Clinical Evidence, and Future Directions. Foods.

[158] Kaur H., Kaur G., Ali S.A. (2022). Dairy-Based Probiotic-Fermented Functional Foods: An Update on Their Health-Promoting Properties. Fermentation.

[159] Saleem G.N., Gu R., Qu H., Bahar Khaskheli G., Rashid Rajput I., Qasim M., Chen X. (2024). Therapeutic Potential of Popular Fermented Dairy Products and Its Benefits on Human Health. Front. Nutr..

[160] Laiño J., Villena J., Kanmani P., Kitazawa H. (2016). Immunoregulatory Effects Triggered by Lactic Acid Bacteria Exopolysaccharides: New Insights into Molecular Interactions with Host Cells. Microorganisms.

[161] Miyoshi Y., Saika A., Nagatake T., Matsunaga A., Kunisawa J., Katakura Y., Yamasaki-Yashiki S. (2021). Mechanisms Underlying Enhanced IgA Production in Peyer’s Patch Cells by Membrane Vesicles Derived from Lactobacillus Sakei. Biosci. Biotechnol. Biochem..

[162] Carasi P., Racedo S.M., Jacquot C., Romanin D.E., Serradell M.A., Urdaci M.C. (2015). Impact of Kefir Derived Lactobacillus Kefiri on the Mucosal Immune Response and Gut Microbiota. J. Immunol. Res..

[163] Yamane T., Sakamoto T., Nakagaki T., Nakano Y. (2018). Lactic Acid Bacteria from Kefir Increase Cytotoxicity of Natural Killer Cells to Tumor Cells. Foods.

[164] Paul A.K., Lim C.L., Apu M.A.I., Dolma K.G., Gupta M., de Lourdes Pereira M., Wilairatana P., Rahmatullah M., Wiart C., Nissapatorn V. (2023). Are Fermented Foods Effective against Inflammatory Diseases?. Int. J. Environ. Res. Public Health.

[165] Kim J., Choi K.-B., Park J.H., Kim K.H. (2019). Metabolite Profile Changes and Increased Antioxidative and Antiinflammatory Activities of Mixed Vegetables after Fermentation by Lactobacillus Plantarum. PLoS ONE.

[166] Oliviero T., Verkerk R., Dekker M. (2018). Isothiocyanates from Brassica Vegetables—Effects of Processing, Cooking, Mastication, and Digestion. Mol. Nutr. Food Res..

[167] Galena A.E., Chai J., Zhang J., Bednarzyk M., Perez D., Ochrietor J.D., Jahan-Mihan A., Arikawa A.Y. (2022). The Effects of Fermented Vegetable Consumption on the Composition of the Intestinal Microbiota and Levels of Inflammatory Markers in Women: A Pilot and Feasibility Study. PLoS ONE.

[168] Hamadou M. (2025). Bioactive Peptides and Metabolic Health: A Mechanistic Review of the Impact on Insulin Sensitivity, Lipid Profiles, and Inflammation. Appl. Food Res..

[169] Ma Z.F., Fu C., Lee Y.Y. (2025). The Modulatory Role of Bioactive Compounds in Functional Foods on Inflammation and Metabolic Pathways in Chronic Diseases. Foods.

[170] Chan M., Larsen N., Baxter H., Jespersen L., Ekinci E.I., Howell K. (2024). The Impact of Botanical Fermented Foods on Metabolic Syndrome and Type 2 Diabetes: A Systematic Review of Randomised Controlled Trials. Nutr. Res. Rev..

[171] Bellikci-Koyu E., Sarer-Yurekli B.P., Karagozlu C., Aydin-Kose F., Ozgen A.G., Buyuktuncer Z. (2022). Probiotic Kefir Consumption Improves Serum Apolipoprotein A1 Levels in Metabolic Syndrome Patients: A Randomized Controlled Clinical Trial. Nutr. Res..

[172] Lange E., Pałkowska-Goździk E., Kęszycka P. (2024). The Influence of Various Types of Functional Bread on Postprandial Glycemia in Healthy Adults. Appl. Sci..

[173] Gil-Cardoso K., Saldaña G., Luengo E., Pastor J., Virto R., Alcaide-Hidalgo J.M., del Bas J.M., Arola L., Caimari A. (2021). Consumption of Sourdough Breads Improves Postprandial Glucose Response and Produces Sourdough-Specific Effects on Biochemical and Inflammatory Parameters and Mineral Absorption. J. Agric. Food Chem..

[174] Stamataki N.S., Yanni A.E., Karathanos V.T. (2017). Bread Making Technology Influences Postprandial Glucose Response: A Review of the Clinical Evidence. Br. J. Nutr..

[175] Biçer Y., Telli A.E., Turkal G., Telli N., Uçar G. (2025). From Raw to Fermented: Uncovering the Microbial Wealth of Dairy. Fermentation.

[176] Tamang J.P., Cotter P.D., Endo A., Han N.S., Kort R., Liu S.Q., Mayo B., Westerik N., Hutkins R. (2020). Fermented Foods in a Global Age: East Meets West. Compr. Rev. Food Sci. Food Saf..

[177] Tlais A.Z.A., Kanwal S., Filannino P., Acin Albiac M., Gobbetti M., Di Cagno R. (2022). Effect of Sequential or Ternary Starters-Assisted Fermentation on the Phenolic and Glucosinolate Profiles of Sauerkraut in Comparison with Spontaneous Fermentation. Food Res. Int..

[178] Kołożyn-Krajewska D., Dolatowski Z. (2008). Tradycyjne i Regionalne Technologie oraz Produkty w Żywieniu Człowieka: Monografia.

[179] Chourasia R., Chiring Phukon L., Abedin M.M., Padhi S., Singh S.P., Rai A.K. (2023). Bioactive Peptides in Fermented Foods and Their Application: A Critical Review. Syst. Microbiol. Biomanufacturing.

[180] Is There Too Much Salt in Lacto-Fermentations?. https://revolutionfermentation.com/en/blogs/fermented-vegetables/too-much-salt-lacto-fermentation/.

[181] Liu X., Ma J., Fan G. (2023). Microbiological Safety and Quality of Fermented Products. Foods.

[182] Dees J. (2019). Fermented Foods Part 2: The Joys and (Few) Risks of At-Home Fermentation. Joyf. Microbe.

[183] Shi H., An F., Lin H., Li M., Wu J., Wu R. (2022). Advances in Fermented Foods Revealed by Multi-Omics: A New Direction toward Precisely Clarifying the Roles of Microorganisms. Front. Microbiol..

